# Draft Genome Sequence of “*Candidatus* Bathyarchaeota” Archaeon BE326-BA-RLH, an Uncultured Denitrifier and Putative Anaerobic Methanotroph from South Africa’s Deep Continental Biosphere

**DOI:** 10.1128/MRA.01295-18

**Published:** 2018-11-21

**Authors:** Rachel L. Harris, Maggie C. Y. Lau, Andreia Cadar, Douglas H. Bartlett, Errol Cason, Esta van Heerden, Tullis C. Onstott

**Affiliations:** a Department of Geosciences, Princeton University, Princeton, New Jersey, USA; b Program in Bioinformatics, Thomas H. Gosnell School of Life Sciences, Rochester Institute of Technology, Rochester, New York, USA; c Marine Biology Research Division, Scripps Institution of Oceanography, University of California at San Diego, La Jolla, California, USA; d Department of Microbial, Biochemical, and Food Biotechnology, University of the Free State, Bloemfontein, South Africa; e Biosaense Solutions, Bloemfontein, South Africa; University of Maryland School of Medicine

## Abstract

Metagenomic sequencing of fracture fluid from South Africa recovered a nearly complete “*Candidatus* Bathyarchaeota” archaeon genome. The metagenome-assembled genome of BE326-BA-RLH contains genes involved in methane metabolism and dissimilatory nitrate reduction. This study presents the first genomic evidence for potential anaerobic methane oxidation in the phylum “*Ca.* Bathyarchaeota.”

## Abstract (precis)

Metagenomic sequencing of fracture fluid from South Africa recovered a nearly complete “*Candidatus* Bathyarchaeota” archaeon genome. The metagenome-assembled genome of BE326-BA-RLH contains genes involved in methane metabolism and dissimilatory nitrate reduction.

## ANNOUNCEMENT

The uncultured “*Candidatus* Bathyarchaeota” is a deeply branching and diverse phylum of deep-biosphere inhabitants whose recently inferred role in methanogenesis supports an early evolution of biogenic methane cycling (1, 2). We report here a near-complete genome sequence of “*Ca.* Bathyarchaeota” archaeon BE326-BA-RLH assembled from metagenomic data obtained from a subsurface chemolithoautotrophic microbial ecosystem (SLiME) (3) in oligotrophic fracture fluid (33.2°C; pH 7.5; reduction potential [pe], –0.48) from 1.34 km below land surface (kmbls) at the Beatrix Gold Mine in South Africa. The genomic DNA (gDNA) was extracted following established procedures (4, 5). The metagenomic library was prepared using the PrepX DNA library kit and an automated Apollo 324 system (WaferGen Biosystems, Inc., Fremont, CA). Paired-end (2 × 100-nucleotide [nt]) metagenomic sequencing was performed at the Marine Biological Laboratory (Woods Hole, MA) using the HiSeq 2000 platform (Illumina, Inc., San Diego, CA).

Tools on the Galaxy Web platform at Princeton University (https://usegalaxy.org) (6) were used to quality filter 43,742,580 reads. Filter FASTQ v.1.1.1 removed reads for which 90% of the bases had a Phred quality score of <30. Remove Sequencing Artifacts v.1.0.1 removed homopolymers. Trim Galore! v.0.4.3.1 removed reads that matched the Illumina universal adapter sequence anywhere with a maximum error rate of 0.1, a match time of 1 nt, and a minimum overlap length of 20 nt, including ambiguous bases (Ns) as matches. Trim v.0.0.1 removed 5 nt from the 3′ end and then reads shorter than 50 nt and those containing Ns. The resulting 34,820,059 reads were assembled using SPAdes v.3.11.0 (–meta option) (7). Scaffolds were binned using MetaBAT v.2.11.2 (8). Reads were mapped back to the metagenome assembly using Bowtie2 v.2.3.2 (default –very-sensitive mode options) (9).

A near-complete (estimated at 89.79%) low-contamination (3.74%) bin with 0% strain heterogeneity was identified as an unknown archaeon using CheckM v.1.0.7 (10). Metagenome reads that mapped to the scaffolds in this initial bin were reassembled using SPAdes v.3.11.0 (default option). The resulting draft genome (2.09 Mb, 44.9% GC content) comprised 227 contigs with an *N*_50_ length of 14,564 bp (Table 1). Protein-encoding genes were identified using Prodigal v.2.6.3 (11) and annotated using NCBI BLAST v.2.2.29+ (12), Prokka v.1.13 (13), GraftM v.0.11.1 (14), and HHpred v.3.0.0beta (15). The final draft genome sequence of BE326-BA-RLH has an 86% coding density and contains 23 30S and 29 50S ribosomal proteins, a single copy of 16S, 23S, and 5S rRNA genes, and 91 tRNA genes. A BLASTn search determined that the 16S rRNA gene (1,139 bp) shares 97% similarity to uncultured “*Ca.* Bathyarchaeota” (GenBank accession numbers EU559699, EU155992, and EU155991) and 87% and 86% similarity to “*Ca.* Bathyarchaeota” methanogens BA2 (GenBank accession number LIHK01000010) and BA1 (GenBank accession number LIHJ01000085), respectively (1).

**TABLE 1:** Statistics summary of methane-metabolizing “*Ca.* Bathyarchaeota” genomes

Statistic	BE326-BA-RLH (this study)	BA1^*a*^	BA2^*a*^
Completeness (%)	89.8^*b*^	91.6	93.8
Contamination (%)	3.7	2.8	3.7
Total length (bp)	2,097,091	1,931,714	1,455,689
GC content (%)	44.9	47.1	44.2
No. of contigs	227	96	58
*N*_50_ value of contigs (bp)	14,564^*c*^	32,677	43,519
No. of coding sequences	2,229	2,403	1,761
Coding density (%)	86.0	80.8	83.6
Avg coverage (×)	21.1	35.8	49.8
Relative abundance (%)	0.36^*d*^	0.92	1.03

a Source: Evans et al. (1).

b Based on lineage-specific marker genes determined via CheckM (9).

c Calculated from Prodigal (10).

d Estimated from the percentage of reads mapped back to the metagenome assembly.

BE326-BA-RLH encodes proteins for hydrogenotrophic, acetoclastic, and methylotrophic methanogenesis and for carbon fixation via the reductive acetyl-CoA pathway. A partial methyl-coenzyme M reductase subunit A (*mcrA*) sequence was identified in a 265-bp-long contig in the metagenome assembly, which shared 70% identity to an amino acid sequence of an uncultured archaeon (GenBank accession number AGA20295) based on a BLASTp search. A maximum likelihood (ML) tree was constructed using RAxML v.8.2.11 (PROTGAMMAILGF mode) (16) for this and for 175 *mcrA* protein sequences obtained from GenBank. The ML tree placed this *mcrA* as a deep branch rooting the “*Ca.* Bathyarchaeota” clade. The GC content and tetranucleotide frequency variance of this *mcrA* were consistent with those of BE326-BA-RLH, but the sequence was too short to be added to the bin.

BE326-BA-RLH contains genes encoding periplasmic nitrate reductase (*narH*) and nitrite reductase (*nrfHA*). Formate dehydrogenase (*fdhAD*) was found adjacent to tungsten-containing formylmethanofuran dehydrogenase (*fwdDACB*), suggesting that formate may serve as a possible electron shuttle between reverse methanogenesis and denitrification. To our knowledge, BE326-BA-RLH is the first described “*Ca.* Bathyarchaeota” genome encoding genes that may couple anaerobic methane oxidation (AOM) to known oxidants. This study provides further support showing that members of the “*Ca.* Bathyarchaeota” may perform AOM (1, 17, 18).

### Data availability.

The BE326 BH2 whole-metagenome shotgun and draft genome sequences of “*Ca.* Bathyarchaeota” BE326-BA-RLH have been deposited at NCBI GenBank under the accession numbers QZGF00000000 (SRA number SRR7867194) and QYYE00000000 (SRA number SRR7866305), respectively. The version described in this paper is version number QYYE01000000.

## References

[1] Evans PN, Parks DH, Chadwick GL, Robbins SJ, Orphan VJ, Golding SD, Tyson GW 2015 Methane metabolism in the archaeal phylum Bathyarchaeota revealed by genome-centric metagenomics. Science 350:434–438. doi:10.1126/science.aac7745. 26494757

[2] Lloyd K 2015 Beyond known methanogens. Science 350:384. doi:10.1126/science.aad4066. 26494746

[3] Stevens TO, McKinley JP 1995 Lithoautotrophic microbial ecosystems in deep basalt aquifers. Science 270:450–455. doi:10.1126/science.270.5235.450.

[4] Lau MCY, Cameron C, Magnabosco C, Brown CT, Schilkey F, Grim S, Hendrickson S, Pullin M, Lollar BS, van Heerden E, Kieft TL, Onstott TC 2014 Phylogeny and phylogeography of functional genes shared among seven terrestrial subsurface metagenomes reveal N-cycling and microbial evolutionary relationships. Front Microbiol 5:53. doi:10.3389/fmicb.2014.00531. 25400621 PMC4215791

[5] Magnabosco C, Tekere M, Lau MCY, Linage B, Kuloyo O, Erasmus M, Cason E, van Heerden E, Borgonie G, Kieft TL, Olivier J, Onstott TC 2014 Comparisons of the composition and biogeographic distribution of the bacterial communities occupying South African thermal springs with those inhabiting deep subsurface fracture water. Front Microbiol 5:679. doi:10.3389/fmicb.2014.00679. 25566203 PMC4269199

[6] Afgan E, Baker D, Batut B, Van Den Beek M, Bouvier D, Ech M, Chilton J, Clements D, Coraor N, Grüning BA, Guerler A, Hillman-Jackson J, Hiltemann S, Jalili V, Rasche H, Soranzo N, Goecks J, Taylor J, Nekrutenko A, Blankenberg D 2018 The Galaxy platform for accessible, reproducible and collaborative biomedical analyses: 2018 update. Nucleic Acids Res 46:W537–W544. doi:10.1093/nar/gky379. 29790989 PMC6030816

[7] Bankevich A, Nurk S, Antipov D, Gurevich AA, Dvorkin M, Kulikov AS, Lesin VM, Nikolenko SI, Pham S, Prjibelski AD, Pyshkin A V., Sirotkin A V., Vyahhi N, Tesler G, Alekseyev MA, Pevzner PA 2012 SPAdes: a new genome assembly algorithm and its applications to single-cell sequencing. J Comput Biol 19:455–477. doi:10.1089/cmb.2012.0021. 22506599 PMC3342519

[8] Kang DD, Froula J, Egan R, Wang Z 2015 MetaBAT, an efficient tool for accurately reconstructing single genomes from complex microbial communities. PeerJ 3:e1165. doi:10.7717/peerj.1165. 26336640 PMC4556158

[9] Langmead B, Salzberg SL 2012 Fast-gapped read alignment with Bowtie 2. Nat. Methods 9:357–359. doi:10.1038/NMETH.1923. 22388286 PMC3322381

[10] Parks DH, Imelfort M, Skennerton CT, Hugenholtz P, Tyson GW 2015 CheckM: assessing the quality of microbial genomes recovered from isolates, single cells, and metagenomes. Genome Res 25:1043–1055. doi:10.1101/gr.186072.114. 25977477 PMC4484387

[11] Hyatt D, Chen GL, LoCascio PF, Land ML, Larimer FW, Hauser LJ 2010 Prodigal: prokaryotic gene recognition and translation initiation site identification. BMC Bioinformatics 11:119. doi:10.1186/1471-2105-11-119. 20211023 PMC2848648

[12] Camacho C, Coulouris G, Avagyan V, Ma N, Papadopoulos J, Bealer K, Madden TL 2009 BLAST+: architecture and applications. BMC Bioinformatics 10:421. doi:10.1186/1471-2105-10-421. 20003500 PMC2803857

[13] Seemann T 2014 Prokka: rapid prokaryotic genome annotation. Bioinformatics 30:2068–2069. doi:10.1093/bioinformatics/btu153. 24642063

[14] Boyd JA, Woodcroft BJ, Tyson GW 2018 GraftM: a tool for scalable, phylogenetically informed classification of genes within metagenomes. Nucleic Acids Res 46:e59. doi:10.1093/nar/gky174. 29562347 PMC6007438

[15] Söding J, Biegert A, Lupas AN 2005 The HHpred interactive server for protein homology detection and structure prediction. Nucleic Acids Res 33:W244–W248. doi:10.1093/nar/gki408. 15980461 PMC1160169

[16] Stamatakis A 2014 RAxML version 8: a tool for phylogenetic analysis and post-analysis of large phylogenies. Bioinformatics 30:1312–1313. doi:10.1093/bioinformatics/btu033. 24451623 PMC3998144

[17] Biddle JF, Lipp JS, Lever MA, Lloyd KG, Sørensen KB, Anderson R, Fredricks HF, Elvert M, Kelly TJ, Schrag DP, Sogin ML, Brenchley JE, Teske A, House CH, Hinrichs K-U 2006 Heterotrophic Archaea dominate sedimentary subsurface ecosystems off Peru. Proc Natl Acad Sci U S A 103:3846–3851. doi:10.1073/pnas.0600035103. 16505362 PMC1533785

[18] Lever MA 2016 A new era of methanogenesis research. Trends Microbiol 24:84–86. doi:10.1016/j.tim.2015.12.005. 26765541

